# Firearms and the incidence of arrest among respondents to domestic violence restraining orders

**DOI:** 10.1186/s40621-015-0047-2

**Published:** 2015-06-23

**Authors:** Garen J. Wintemute, Shannon Frattaroli, Mona A. Wright, Barbara E. Claire, Katherine A. Vittes, Daniel W. Webster

**Affiliations:** 1 Violence Prevention Research Program, Department of Emergency Medicine School of Medicine, University of California, Davis; 2315 Stockton Blvd, Sacramento, CA 95817 USA; 2 Center for Gun Policy and Research, Johns Hopkins Bloomberg School of Public Health, Baltimore, MD USA

**Keywords:** MeSH terms, Domestic violence, Firearms, Crime, Public policy, Law enforcement, Non MeSH, Intimate partner violence

## Abstract

**Background:**

Persons subject to domestic violence restraining orders (DVROs), known as respondents, are generally prohibited from possessing firearms. Efforts to enforce that prohibition have not been evaluated. The study objective was to determine whether associations exist between risk of incident arrest among DVRO respondents and 1) respondents’ access to firearms, and 2) law enforcement recovery of firearms from respondents with access to them.

**Methods:**

This was an observational study of 2,972 DVRO respondents in San Mateo County, California, 525 of whom were linked to firearms by standardized screening procedures. Enrollment occurred from May 2007 to June 2010 and follow-up through September 2010. Follow-up began when DVROs were served (or when issued if no date of service was available); median duration was 689 days. Principal exposures were access to firearms and, for subjects with access to firearms whose DVROs were served, contact by law enforcement personnel to recover those firearms. Main outcome measures were 1) incidence of arrest; 2) relative risk for arrest, adjusted for age, sex, prior criminal history, and duration of follow-up, assessed using logistic regression.

**Results:**

Respondents linked to firearms were older than others and were more likely to have a history of prior arrest (49.7 % and 37.3 %, p < 0.0001). The incidence of arrest was 20.6 % for respondents linked to firearms and 21.1 % for others (p = 0.78). In multivariate models, access to firearms was associated with a modest, generally not statistically significant, decrease in risk for incident arrest. Among respondents who were linked to firearms and whose restraining orders were served, no statistically significant association existed between firearm recovery and risk for incident arrest.

**Conclusions:**

In this small study of DVRO respondents, findings are inconclusive for an association between access to firearms or firearm recovery and risk of incident arrest. Controlled trials on larger populations are indicated.

**Electronic supplementary material:**

The online version of this article (doi:10.1186/s40621-015-0047-2) contains supplementary material, which is available to authorized users.

## Background

Intimate partner violence (IPV) is a significant public health problem. National Crime Victimization Survey data suggest that during the 10 years 2004–2013, there were on average nearly 950,000 instances of intimate partner violence annually in the United States (Bureau of Justice Statistics). The 2011 National Intimate Partner and Sexual Violence Survey reports that 22.3 % of women and 14.0 % of men in the United States have experienced “severe physical violence” perpetrated by an intimate partner at some time in their lives (Breiding et al. 2014). At least 992 women and 245 men were murdered by their intimate partners in the United States in 2013 (Federal Bureau of Investigation 2014).

Firearms play an important role in IPV. In 2008, firearms were used in 53.0 % of female and 41.9 % of male intimate partner homicides (Cooper and Smith 2011). Access to firearms by male abusers is associated with a greater than 7-fold increase in risk for homicide for their intimate partners (Campbell et al. 2003). Approximately 1 in 30 women reports that her intimate partner has threatened her with a firearm (Tjaden and Thoennes 2000). Among women in shelters in California who came from households with firearms, 64.5 % reported that their partner used a firearm to threaten or assault them (Sorenson and Wiebe 2004). In large urban counties, 6.2 % of felony prosecutions for IPV involved an assault with a firearm (Smith and Farole 2009).

Domestic violence restraining orders (DVROs), a widely accepted violence prevention measure, are available in every state (DeJong and Burgess-Proctor 2006); more than 1 million may be issued annually (Moracco et al. 2010). Well described as “an institutionalized form of exposure reduction,”(Dugan et al. 2003, pg 174) they provide enforceable judicial sanction to the separation of an abused person from her abuser. Such sanction is appropriate, in that serious and violent criminal behavior is very common among persons subject to DVROs (Jordan et al. 2010; Moracco et al. 2010; Vittes and Sorenson 2006), and a woman’s risk for IPV is highest immediately after she attempts to leave an abusive partner (Campbell et al. 2003; Wilson and Daly 1993).

Persons subject to DVROs, known as respondents, are generally prohibited from purchasing or possessing firearms. Although the federal prohibition is limited to orders issued at hearings where both parties are present, many states include temporary DVROs issued without a hearing. Increasingly, states authorize or require courts to order DVRO respondents to relinquish their firearms (Frattaroli 2009; Zeoli and Bonomi 2015). California, where this study was conducted, requires respondents to surrender their firearms to law enforcement or sell them to a licensed retailer within 24 hours after the order is served, provide evidence of compliance to the court within 48 hours, and surrender firearms immediately if instructed to do so by a law enforcement officer (California Family Code).

Population-based ecological studies suggest that statutes prohibiting DVRO respondents from purchasing or possessing firearms are effective, though statutes allowing law enforcement officers to recover firearms at scenes of domestic violence are not (Vigdor and Mercy 2003, 2006; Zeoli and Webster 2010). Such studies do not present data for affected individuals, however. An analysis of individual-level data found that simply requiring respondents to surrender firearms did not reduce the proportion of respondents who possessed firearms or the rate of firearm-related IPV (Moracco et al. 2006).

To our knowledge, no studies have assessed the association between firearm access among individual DVRO respondents, or the effect of enforcing the DVRO firearm prohibition by recovering firearms from respondents who have them, on respondents’ risk for subsequent criminal activity (Klein AR 2006). We previously reported a process assessment of a pilot initiative in California during which DVRO respondents were screened for access to firearms and an effort was made to enforce the firearm prohibition (Wintemute et al. 2014). San Mateo County accounted for 91.3 % of screened respondents and provided their identifying information. (The other participating county did not retain respondent identifiers.) For San Mateo County, we examine here the association between respondents’ risk for arrest following issuance of a DVRO and 1) access to firearms, and 2) recovery of firearms from respondents with access to them. Our hypothesis was that recovery of firearms would be associated with a reduced risk of incident arrest, including for domestic violence and other violent and firearm-related crimes.

## Methods

### Study site and population

San Mateo County had a population of 718,451 in 2010: 42.3 % White, 25.4 % Hispanic/Latino, 24.8 % Asian, 2.8 % Black, and 4.7 % other, with 98.1 % living in Census-defined urban areas (State and County QuickFacts [database on the Internet]. Washington, DC: U.S. Census Bureau 2011). The 2007–2011 median household income, $87,633, was 42.2 % higher than that for California as a whole (State and County QuickFacts [database on the Internet]. Washington, DC: U.S. Census Bureau 2011). The county reported 2,766 domestic violence calls for assistance in 2010 (Criminal Justice Statistics Center). The county sheriff had primary jurisdiction over unincorporated areas of the county; 22 other jurisdictions had their own law enforcement agencies.

The study population comprised all respondents to DVROs issued between May 2007 and June 2010. Two sheriff’s detectives, who worked solely on the initiative and were responsible for the entire county, screened respondents for access to firearms following standardized procedures. After receiving DVROs from the county court, detectives reviewed petitioner declarations and other court documents. (The DVRO application form asked whether respondents had access to firearms, and victim advocates encouraged reporting. Photographs of representative firearms were available.) Detectives also queried California’s Automated Firearms System (AFS), which recorded all handgun purchases processed by licensed retailers and all denied purchases, both since 1996; assault weapon registrations; and concealed weapon permit applications. They searched the Armed and Prohibited Persons System (a registry of prohibited persons believed to own firearms) and other databases. When these procedures linked respondents to firearms, detectives often interviewed petitioners. Information on firearm access ranged from specific knowledge, based on ownership records in AFS or a petitioner’s detailed eyewitness report, to a petitioner’s indication that a respondent had mentioned having access to a firearm.

### Description of the intervention

When petitioners had their DVROs served by private parties, the detectives usually received notice after the fact when proof of service was received by the court. If the respondent was linked to firearms, the detectives contacted him or her to explain the prohibition. If the respondent acknowledged having firearms, the detectives, sheriff’s deputies, or local police officers took custody of them or facilitated their sale to a retailer.

When petitioners requested that law enforcement personnel serve their DVROs, the standard practice was to have this done by deputies in the civil division of the sheriff’s office. Detectives advised the deputies beforehand whether screening had linked the respondent to firearms. Civil deputies explained the prohibition to the respondent when serving the order, but their scope of practice did not allow them to recover firearms. If there were firearms at the scene, deputies were expected to wait for a detective or another officer to arrive and recover them. Given their heavy workloads, waiting was not always possible. Service by the detectives themselves was generally limited to respondents who were in custody.

Respondents linked to firearms sometimes denied possessing them. Search warrants were generally not available before January 2010, near the end of the study period, when new state statutes took effect (California Statutes of 2009). Respondents could be asked to certify under penalty of perjury that they had no firearms, but they could not be compelled to do so and faced no penalty for refusing. Corroborating evidence for such certifications was not required until early 2009.

### Data and analysis

The San Mateo County Sheriff’s Office provided us with respondents’ identifiers (name, birthdate, Social Security and driver’s license numbers), firearm screening results, and data on DVRO service and firearm recovery. We requested criminal records from the California Department of Justice (CalDOJ) and required concordance on name, birthdate, and either Social Security or driver’s license number for a match. We received the records electronically and extracted data on all charges for which respondents had ever been arrested or convicted. Offenses were classified as involving neither firearms nor violence, as domestic violence, or as other violent or firearm-related offenses (the records did not always distinguish between violent offenses that involved firearms and those that did not). We reviewed prior convictions to identify respondents who were already prohibited from possessing firearms under federal or California law. Prohibited persons were assigned to mutually-exclusive categories by prohibition type, hierarchically: felony conviction > domestic violence misdemeanor conviction > other violent misdemeanor conviction.

Our outcome measure was an arrest during follow-up, for any offense and by type of offense. Follow-up began when restraining orders were served (or when issued if no date of service was available), and ended on September 7, 2010, the date of our records request.

The prevalence and incidence of arrest were expressed as counts and proportions of respondents. Significance of differences was assessed using the Pearson chi-squared test. Continuous variables were summarized by medians with interquartile ranges (IQRs); significance of differences was assessed using the Wilcoxon test.

We used logistic regression to determine whether characteristics of interest were associated with risk of an incident arrest, expressing results as odds ratios (ORs) with 95 % confidence intervals (CIs). Continuous variables other than duration of follow-up were stratified for analysis. Variables for interactions between firearm recovery and age, number of prior arrest charges, and time since most recent arrest were used to determine whether the effect of firearm recovery varied across subsets of respondents.

Our criminal records review determined that 82 (3.4 %) of 2,447 respondents who had not screened positive for firearms had prior arrests for firearm-related crimes. In the main analysis, these respondents were classified according to their screening results. We conducted a sensitivity analysis with these respondents classified as having access to firearms.

*P* < 0.05 was taken as the threshold for statistical significance. Analyses were performed using SAS version 9.3 (SAS Institute Inc) for Windows.

## Results

Detectives screened 2,972 DVRO respondents during the study period, linking 525 (17.7 %) to firearms. DVROs were served on 56.4 % of respondents (1,677 of 2,972), including 68.8 % of those linked to firearms (361 of 525) and 53.8 % of those not linked to firearms (1,316 of 2,447) (p < 0.0001). Firearms were recovered from 33.0 % of respondents linked to firearms whose orders were served (119 of 361).

### Characteristics of the study population

Respondents linked to firearms were more likely than others to be male (91.6 % and 80.0 %, respectively, p < 0.0001), were older, and were more likely to have a history of prior arrest (49.7 % and 37.3 %, respectively, p < 0.0001) (Table 1). Most respondents with any arrest history in both groups had 6 or more prior arrest charges, had been arrested for domestic violence and other firearm-related or violent offenses, and had been arrested within approximately a year of their DVROs.

**Table 1:** Respondents’ personal characteristics by presence or absence of linkage to firearms

Characteristic	Not Linked to Firearms	Linked to Firearms	P value
	(n = 2,447)	(n = 525)	
Sex, No. (%)			
Male	1,958 (80.0)	481 (91.6)	<0.0001
Female	489 (20.0)	44 (8.4)	
Age, No. (%)^a^			
≤24	379 (15.9)	39 (7.4)	<0.0001
25-34	684 (28.7)	123 (23.5)	
35-44	702 (29.5)	148 (28.2)	
≥45	617 (25.9)	214 (40.8)	
median (IQR)	36 (27–45)	41 (31–49)	<0.0001
Arrest history, No. (%)			
None	1,535 (62.7)	264 (50.3)	<0.0001
Any offense	912 (37.3)	261 (49.7)	
Arrest charges for specific offenses, No. (%)			
Offenses not involving violence or firearms	778 (31.8)	221 (42.1)	<0.0001
Domestic violence	466 (19.0)	133 (25.3)	<0.0001
Other violent or firearm-related offenses	574 (23.5)	173 (33.0)	<0.0001
Number of prior arrest charges per respondent,^b^ median (IQR)			
Any offense	7 (2.5-22)	6 (2–13)	0.008
Offenses not involving violence or firearms	5 (1–14)	3 (1–9)	0.003
Domestic violence	1 (0–2)	1 (0–1)	0.50
Other violent or firearm-related offenses	2 (1–5)	2 (1–4)	0.33
Years from most recent arrest to date of restraining order,^b^ No. (%)			
0-5	756 (82.9)	197 (75.5)	0.016
6+	156 (17.1)	64 (24.5)	
median (IQR)	0.7 (0.06-2.9)	1.1(0.07-4.9)	
Pre-existing firearms prohibition,^b^ No. (%)			
Y	461 (50.5)	117 (44.8)	0.11
N	451 (49.6)	144 (55.2)	

Note. IQR = interquartile range

^a^Age is missing for 66 individuals

^b^Results are for respondents with prior arrests

Many respondents were prohibited from possessing firearms prior to becoming subject to DVROs, including 44.8 % of those with any arrest history who were linked to firearms (Table 1). Of all 578 prohibited persons, 472 (81.7 %) were felons, 62 (10.7 %) had domestic violence misdemeanor convictions, and 44 (7.6 %) had convictions for other violent misdemeanors within the preceding 10 years (the last group is prohibited under California but not federal law). Among the 361 respondents who were linked to firearms and whose orders were served, those from whom firearms were recovered were older than others [median (IQR) 44 (39–51) and 41 (30–49), respectively, p = 0.0003]. Though they were nearly as likely as others to have been arrested previously, they had less extensive prior arrest histories (Additional file 1: Table S1). Firearms were less likely to be recovered from respondents who were already prohibited persons than from others (11 of 79 (13.9 %) and 108 of 282 (38.3 %), respectively, p = <0.0001).

### Incidence of arrest

The median duration of follow-up was 689 days (IQR 389–972 days), during which 625 respondents (21.0 %) were arrested at least once. Men, younger respondents, and particularly those with a prior history of arrest were at increased risk of incident arrest (Table 2). Among previously-arrested respondents, risk of incident arrest was further increased for those who had multiple prior arrest charges, had been arrested within the 5 years preceding their restraining orders, or were already prohibited persons. Restraining order service was associated with a moderate increase in risk of arrest that was not always statistically significant.

**Table 2:** Incidence of arrest among respondents by personal characteristics, restraining order service, linkage to firearms, and firearm recovery

Characteristic	Respondents with new arrests^a^, no. (%)							
	Any offense	P value	Offenses not involving violence or firearms	P value	Domestic violence	P value	Other violent or firearm-related offenses	P value
All respondents (n = 2,972)	625 (21.0)		507 (17.1)		238 (8.0)		229 (7.7)	
Sex								
Male (n = 2,439)	544 (22.3)	0.0003	441 (18.1)	0.0015	209 (8.6)	0.0159	209 (8.6)	0.0002
Female (n = 533)	81 (15.2)		66 (12.4)		29 (5.4) (3.8)		20 (3.8)	
Age^b^, years								
≤24 (n = 418)	107 (25.6)	0.0006	84 (20.1)	0.0008	37 (8.9)	0.1245	41 (9.8)	0.0333
25-34 (n = 807)	198 (24.5)		166 (20.6)		78 (9.7)		73 (9.1)	
35-44 (n = 850)	176 (20.7)		145 (17.1)		69 (8.1)		67 (7.9)	
≥45 (n = 831)	144 (17.3)		112 (13.5)		54 (6.5)		48 (5.8)	
Arrest history								
None (n = 1,799)	68 (3.8)	<0.0001	42 (2.3)	<0.0001	33 (1.8)	<0.0001	15 (0.8)	<0.0001
Any offense (n = 1,173)	557 (47.5)		465 (39.6)		205 (17.5)		214 (18.2)	
Number of prior arrest charges^c^								
Any offense								
1-2 (n = 299)	73 (24.4)	<0.0001	57 (19.1)	<0.0001	23 (7.7)	<0.0001	28 (9.4)	<0.0001
3-9 (n = 378)	152 (40.2)		123 (32.5)		66 (17.5)		47 (12.4)	
10+ (n = 496)	332 (66.9)		285 (57.5)		116 (23.4)		139 (28.0)	
Offenses not involving violence or firearms								
1 (n = 194)	56 (28.9)	<0.0001	41 (21.1)	<0.0001	18 (9.3)	0.0002	21 (10.8)	0.0003
2+ (n = 805)	463 (57.5)		397 (49.3)		169 (21.0)		180 (22.4)	
Domestic violence								
1 (n = 312)	127 (40.7)	0.0027	102 (32.7)	0.0210	55 (17.6)	0.0153	53 (17.0)	0.0322
2+ (n = 287)	152 (53.0)		120 (41.8)		74 (25.8)		69 (24.0)	
Other violent or firearm-related offenses								
1 (n = 229)	104 (45.4)	0.0057	84 (36.7)	0.0061	40 (17.5)	0.4044	32 (14.0)	0.0004
2+ (n = 518)	292 (56.4)		246 (47.5)		104 (20.1)		133 (25.7)	
Time from most recent arrest to date of restraining order^c^, years								
0-5 (n = 953)	499 (52.4)	<0.0001	424 (44.5)	<0.0001	184 (19.3)	0.0006	193 (20.3)	0.0002
6+ (n = 220)	58 (26.7)		41 (18.6)		21 (9.6)		21 (9.6)	
Pre-existing firearms prohibition^c^								
Y (n = 578)	335 (58.0)	<0.0001	288 (49.8)	<0.0001	117 (20.2)	0.0140	130 (22.5)	0.0002
N (n = 595)	222 (37.3)		177 (29.8)		88 (14.8)		84 (14.1)	
Order served								
Y (n = 1,677)	387 (23.1)	0.0018	314 (18.7)	0.0060	162 (9.7)	0.0002	143 (8.5)	0.0559
N (n = 1,295)	238 (18.4)		193 (14.9)		76 (5.9)		86 (6.6)	
Linked to firearms								
Y (n = 525)	108 (20.6)	0.7765	84 (16.0)	0.4770	37 (7.1)	0.3716	41 (7.8)	0.9213
N (n = 2447)	517 (21.1)		423 (17.3)		201 (8.2)		188 (7.7)	
Firearms recovered^d^								
Y (n = 119)	14 (11.8)	0.005	8 (6.7)	0.0027	9 (7.6)	0.9661	6 (5.0)	0.1426
N (n = 242)	59 (24.4)		45 (18.6)		18 (7.4)		23 (9.5)	

^a^A respondent may have arrests for several types of offenses

^b^Age was missing for 66 individuals

^c^Results are for respondents with prior arrests

^d^Results are for 361 respondents who were linked to firearms and whose orders were served

There was no difference in risk of arrest between respondents who were linked to firearms and others (Table 2). Among all respondents with an incident arrest, there was no difference in the incidence of multiple arrests between those who were linked to firearms and others (53.7 % and 56.9 %, respectively, p = 0.55). Among the 361 respondents who were linked to firearms and whose orders had been served, those from whom firearms were recovered had a lower incidence of arrest than others did, for all offenses combined and for those not involving firearms or violence (Table 2). For the subset of these respondents who experienced an incident arrest (n = 73), multiple arrests occurred with similar frequency among those whose firearms were recovered and others (64.3 % and 52.5 %, respectively, p = 0.43).

In bivariate regressions (Additional file 2: Table S2), linkage to firearms and firearm recovery were the only characteristics not associated with risk for incident arrest for all types of offenses. In multivariate models for all respondents (Table 3) and in the sensitivity analysis (Additional file 3: Table S3), respondents’ age and prior arrest history were most strongly associated with risk of arrest, and there was a trend toward a decrease in risk associated with a linkage to firearms. Among respondents who were linked to firearms and whose orders were served (Table 4), risk of arrest was not associated with firearm recovery. Interaction terms were not statistically significant.

**Table 3:** For all subjects, multivariate associations between risk of incident arrest and respondent characteristics, prior criminal history, restraining order service, and linkage to firearms^a^

Order-recovery status and respondent characteristic	Risk of incident arrest							
	Any offense		Offenses not involving violence or firearms		Domestic violence		Other violent or firearm-related offenses	
	OR	P value	OR	P value	OR	P value	OR	P value
	(95 % CI)		(95 % CI)		(95 % CI)		(95 % CI)	
Sex								
Male (n = 2,439)	1.0 (0.7-1.4)	0.8975	1.0 (0.7-1.4)	0.8651	1.1 (0.7-1.7)	0.6028	1.7 (1.0-2.8)	0.0516
Female (n = 533)	Referent		Referent		Referent		Referent	
Age^b^, years								
≤24 (n = 418)	2.7 (1.9-4.0)	<0.0001	2.4 (1.6-3.7)	<0.0001	1.5 (1.0-2.5)	0.1759	2.5 (1.5-4.1)	0.0023
25-34 (n = 807)	1.5 (1.1-2.0)		1.5 (1.1-2.1)		1.3 (0.9-2.0)		1.4 (0.9-2.2)	
35-44 (n = 850)	1.0 (0.7-1.3)		1.0 (0.7-1.4)		1.0 (0.7-1.6)		1.1 (0.8-1.7)	
≥45 (n = 831)	Referent		Referent		Referent		Referent	
Prior arrest charges								
10+ (n = 496)	69.8 (50.0-97.3)	<0.0001	77.1 (52.8-112.5)	<0.0001	16.8 (11.2-25.4)	<0.0001	51.3 (29.4-89.3)	<0.0001
3-9 (n = 378)	22.9 (16.3-32.3)		27.7 (18.7-41.1)		12.5 (8.0-19.6)		18.7 (10.2-34.2)	
1-2 (n = 299)	11.5 (7.9-16.9)		14.3 (9.2-22.3)		5.2 (3.0-9.2)		15.4 (8.0-29.8)	
None (n = 1799)	Referent		Referent		Referent		Referent	
Time from most recent arrest charge to date of restraining order ^c^, years								
0-5 (n = 953)	1.8 (1.2-2.5)	0.0091	2.1(1.4-3.1)	0.0002	1.5 (0.9-2.5)	0.0967	1.5 (0.9-2.5)	0.1360
6+ (n = 220)	Referent		Referent		Referent		Referent	
Order served								
Y (n = 1,677)	1.2 (1.0-1.5)	0.0881	1.2 (1.0-1.6)	0.1213	1.6 (1.2-2.2)	0.0018	1.2 (0.9-1.6)	0.3123
N (n = 1,295)	Referent		Referent		Referent		Referent	
Linked to firearms								
Y (n = 525)	0.8 (0.6-1.1)	0.1069	0.8 (0.6-1.0)	0.0802	0.7 (0.5-1.0)	0.0609	0.9 (0.6-1.4)	0.6634
N (n = 2,447)	Referent		Referent		Referent		Referent	
Follow-up time, per month								
	1.0 (1.0-1.0)	<0.0001	1.0 (1.0-1.1)	<0.0001	1.0 (1.0-1.0)	0.0039	1.0 (1.0-1.0)	<0.0001

^a^Model includes all variables in table

^b^Age was missing for 66 individuals

^c^Results are for respondents with prior arrests. Pre-existing firearm prohibition status and number of prior arrest charges were highly correlated and could not both be entered into the model

**Table 4:** For 361 respondents who were linked to firearms and whose orders were served, multivariate associations between risk of incident arrest and respondent characteristics and firearm recovery^a^

Order-recovery status and respondent characteristic	Risk of Incident Arrest							
	Any Offense		Offenses not involving violence or firearms		Domestic Violence		Other violent or firearm-related offenses^d^	
	OR	P value	OR	P value	OR	P value	OR	P value
	(95 % CI)		(95 % CI)		(95 % CI)		(95 % CI)	
Sex								
Male (n = 334)	10.4 (1.1-99.1)	0.0413	7.2 (0.7-70.8)	0.0915	1.8 (0.2-16.1)	0.5868	-	
Female (n = 27)	Referent		Referent		Referent		-	-
Age^b^, years								
≤24 (n = 24)	3.4 (1.0-11.9)	0.0550	4.3 (1.2-15.9)	0.0228	0.4 (0.0-3.8)	0.5069	1.5 (0.3-6.8)	0.7222
25-34 (n = 75)	1.7 (0.7-3.9)		3.4 (1.4-8.5)		1.0 (0.4-3.0)		1.4 (0.5-4.0)	
35-44 (n = 112)	0.7 (0.3-1.4)		1.3 (0.6-3.2)		0.5 (0.1-1.4)		0.8 (0.3-2.2)	
≥45 (n = 149)	Referent		Referent		Referent		Referent	
Prior arrest charges								
10+ (n = 66)	41.8 (15.7-111.4)	<0.0001	37.4 (11.5-121.1)	<0.0001	26.6 (5.6-125.2)	0.0003	13.3 (4.1-43.5)	<0.0001
3-9 (n = 63)	16.4 (6.1-43.6)		19.7 (5.9-65.6)		17.2 (3.5-83.4)		9.7 (2.8-32.9)	
1-2 (n = 55)	10.2 (3.3-31.6)		15.7 (4.0-60.9)		7.1 (1.1-45.4)		1.3 (0.1-12.4)	
None (n = 177)	Referent		Referent		Referent		Referent	
Time from most recent arrest charge to date of restraining order ^c^, years								
0-5 (n = 318)	2.9 (1.1-7.9)	0.0377	3.0 (0.9-9.9)	0.0680	2.9 (0.6-13.7)	0.1715	4.8 (0.6-38.2)	0.1428
6+ (n = 43)	Referent		Referent		Referent		Referent	
Firearms recovered								
Y (n = 119)	0.5 (0.3-1.2)	0.1164	0.4 (0.2-1.0)	0.0440	1.4 (0.6-3.7)	0.4373	0.7 (0.3-2.0)	0.5475
N (n = 242)	Referent		Referent		Referent		Referent	
Follow-up time, per month								
	1.1 (1.0-1.1)	0.0013	1.1 (1.0-1.1)	0.0038	1.0 (1.0-1.1)	0.2482	1.0 (1.0-1.1)	0.1160

^a^Model includes all variables in table

^b^Age was missing for 1 individual

^c^Results are for respondents with prior arrests. Only results for years from most recent arrest are given. Pre-existing firearm prohibition status and number of prior arrest charges were highly correlated and could not both be entered into the model

d Results are for males only. No female respondents were arrested for offenses of this type

## Discussion

In this population of respondents to DVROs, arrest following service of a restraining order was common, as previous studies have shown (Jordan et al. 2010; Moracco et al. 2010; Vittes and Sorenson 2006; Moracco et al. 2006). Men, younger individuals, and those with more extensive prior criminal histories were at increased risk for an incident arrest. These findings are consistent with those for a wide range of populations, including DVRO respondents, (Jordan et al. 2010) domestic violence offenders generally (Cattaneo and Goodman 2005), and firearm owners (Wintemute et al. 1998; Wright and Wintemute 2010).

Firearm access was associated with a modest, generally not statistically significant, decrease in risk for incident arrest in multivariate models. Among respondents linked to firearms whose restraining orders were served, no statistically significant association with firearm recovery was observed. We did not find that firearm recovery reduced the incidence of violent criminal activity, arguably the goal of the initiative.

The absence of statistical significance results at least partly from the small number of respondents and short follow-up period, which reduced the number of incident arrests below that needed to assign statistical significance even to some large effects. It is instructive to consider other explanations for our findings as well.

The absence of an effect associated with firearm recovery could occur for several reasons related to the nature of the intervention. (1) Since DVROs themselves reduce risk for domestic violence (Carlson et al. 1999; Logan and Walker 2010; Holt et al. 2002; Holt et al. 2003; Kothari et al. 2012), firearm recovery may provide no additional benefit. (2) A benefit specific to firearm-related domestic violence might not be detectable in a study of this size, as firearms are used in a minority of domestic violence events. (3) As arrests are an insensitive measure of domestic violence (Holt et al. 2002; Holt et al. 2003), firearm recovery may produce benefits that we did not capture. Our interviews with petitioners in cases from this study support this possibility (Vittes et al. 2013). (4) Petitioners who know that firearms have been recovered might feel safer as a result and be more willing to contact the police regarding criminal acts by respondents, which might increase the frequency of arrests. (5) Such petitioners might be more likely to continue in their relationships, increasing their exposure to further violence. (6) A subset of respondents, angered by the recovery of firearms might increase their violent behavior and risk of arrest. (7) An overall lack of effect might mask important variation across subsets of respondents (in this small study, we did not detect such variation). 

The lack of an effect could also result from flaws in the design or implementation of the initiative. Enforcement of restraining orders, including their firearms provisions, is generally difficult and often goes undone (Moracco et al. 2010; Holt et al. 2002; Frattaroli and Teret 2006; Seave 2006; Webster et al. 2010; Sorenson and Shen 2005; Attorney General's Task Force on Local Criminal Justice Response to Domestic Violence 2005). In this study, failure to serve DVROs was the leading source of failure to recover firearms from respondents who had them, accounting for almost 40 % of such cases. Previous studies have documented non-service rates ranging from 16 % to 66 % (Sorenson and Shen 2005; Logan et al. 2005; Meloy et al. 1997). Maximizing rates of service in order to maximize firearm recovery may not be beneficial, however; restraining order service was associated with a trend toward an increasing risk of arrest in this study population. This could reflect an adverse effect of restraining order service; selection bias, with orders more likely to be served on individuals at increased risk for future violence; or both.

Service of orders without immediate recovery of firearms may have allowed some respondents to dispose of their firearms temporarily before officers arrived to recover them. Others may have falsely denied possessing firearms. With recent changes in California statute, search warrants may be more readily available in such cases (California Statutes of 2009); CalDOJ no longer accepts uncorroborated claims of non-possession.

Screening for access to firearms would be improved by including records of prior firearm-related criminal activity. Records for rifles and shotguns have been added to AFS since January 2014 (California Statutes of 2011). False-negative results will still occur when no records of respondents’ access to firearms exist and petitioners do not report it.

Respondents with pre-existing firearm prohibitions were numerous and pose a special problem. They have a particular incentive to deny possession or dispose of firearms if given the opportunity; recovery could lead to a felony prosecution for illegal possession. The possibility of the respondent’s incarceration might also deter some petitioners from having DVROs served.

### Limitations

There were important differences between respondents with and without access to firearms and, among respondents with access to firearms, between those from whom firearms were recovered and others—chiefly the lower number of prior arrest charges in the recovery group. Among individuals with firearms, in this study and others (Moracco et al. 2010; Vittes and Sorenson 2006; Wright et al. 1999; Wintemute et al. 1998), the extent of any prior criminal history is an important risk factor for subsequent criminal activity. It is possible that the principal effect of the effort to recover firearms was to force respondents to select themselves into 2 groups—those who would comply with the prohibition and those who would not—that differed for other reasons in their risk of subsequent arrest.

Given the important observed differences between subgroups of respondents, bias due to residual confounding is likely. We do not have data on alcohol abuse, for example, a risk factor for violence (Kellermann et al. 1993; Rivara et al. 1997) that is associated with firearm ownership and risk-taking behavior involving firearms (Wintemute 2011; Nelson et al. 1996).

We have necessarily used arrest, not conviction, as our measure of criminal activity. Final outcomes, known as dispositions, are missing for approximately 46 % of California arrests over the past 10 years (Dolan 2011). Arrest is a common measure of both prevalent and incident offending in criminologic research (Blumstein and Nakamura 2009; Kurlychek et al. 2007; Rosenfeld et al. 2005; Langan and Levin 2002). We do not have data on arrests outside California or on arrests specifically for restraining order violations. Follow-up was for a relatively brief and variable period, and we are not able to address long-term effects of firearm recovery.

DVRO respondents differ substantially from the general population in their level of criminal activity; findings related to firearm ownership in this population are not generalizable.

## Conclusion

Firearm prohibitions related to DVROs are widely supported (Sorenson 2006). Our findings are inconclusive but provide a basis in evidence for larger-scale trials of enforcing firearms prohibitions for DVRO respondents and, by extension, other prohibited groups. Randomized trials would be ideal in order to minimize selection bias and confounding, and studies should be designed so as to determine the mechanism by which any observed effect is achieved.

## Additional files

Additional file 1: Table S1. Personal characteristics by whether or not firearms were recovered, for 361 respondents who were linked to firearms and whose restraining orders were served. Additional file 2: Table S2. Bivariate associations between risk of incident arrest and respondent characteristics, restraining order service, linkage to firearms, and firearm recovery. Additional file 3: Table S3. Sensitivity analysis.
