# Supplementary material for: Firearms and the incidence of arrest among respondents to domestic violence restraining orders
Source: Inj Epidemiol. 2015 Jun 23;2(1):14. doi: 10.1186/s40621-015-0047-2 (PMC5005597; doi:10.1186/s40621-015-0047-2)
Supplement: Additional file 1: Table S1. — Personal characteristics by whether or not firearms were recovered, for 361 respondents who were linked to firearms and whose restraining orders were served. [file 40621_2015_47_MOESM1_ESM.docx]

SupplementalTable 1. Personal characteristics by whether or not firearms were recovered, for 361 respondents who were linked to firearms and whose restraining orders were served

| Characteristic | Firearms  Recovered  (n= 119) | Firearms  Not Recovered  (n= 242) | p Value |
| --- | --- | --- | --- |
| Sex, No. (%) | | | |
| Male | 110 (92.4) | 224 (92.6) | 0.97 |
| Female | 9 (7.6) | 18 (7.4) |  |
| Age, years, No. (%)^a^ | | | |
| ≤24 | 0 (0.0) | 24 (9.9) | 0.0008 |
| 25-34 | 19 (16.1) | 56 (23.1) |  |
| 35-44 | 42 (35.6) | 70 (28.9) |  |
| ≥45 | 57 (48.3) | 92 (38.0) |  |
| median (IQR) | 44 (39-51) | 41 (30-49) | 0.0003 |
| Arrest history, No. (%) | | | |
| None | 62 (52.1) | 115 (47.5) | 0.41 |
| Any offense | 57 (47.9) | 127 (52.5) |  |
| Number of prior arrest charges per respondent,^b^ median (IQR) | | | |
| Any offense | 3 (2-7) | 7 (2-21) | 0.002 |
| Offenses not involving violence or firearms | 1 (0-3) | 4 (1-14) | 0.0001 |
| Domestic violence | 0 (0-1) | 1 (0-1) | 0.38 |
| Other violent or firearm-related offenses | 1 (0-2) | 1 (0-5) | 0.49 |
| Years from most recent arrest to date of restraining order,^b^ No. (%) | | | |
| 0-5 | 105 (88.2) | 213 (88.0) | 0.95 |
| 6+ | 14 (11.8) | 29 (12.0) |  |
| median (IQR) | 0 (0-0.2) | 0.01 (0-1.6) | 0.14 |
| Pre-existing firearms prohibition,^b^ No. (%) | | | |
| Y | 11 (9.2) | 68 (28.1) | <0.0001 |
| N | 108 (90.8) | 174 (71.9) |  |

*Note*. IQR=interquartile range.

^a^ Age is missing for 1 individual.

^b^ Results are for respondents with prior arrests.
