# Supplementary material for: Firearms and the incidence of arrest among respondents to domestic violence restraining orders
Source: Inj Epidemiol. 2015 Jun 23;2(1):14. doi: 10.1186/s40621-015-0047-2 (PMC5005597; doi:10.1186/s40621-015-0047-2)
Supplement: Additional file 2: Table S2. — Bivariate associations between risk of incident arrest and respondent characteristics, restraining order service, linkage to firearms, and firearm recovery. [file 40621_2015_47_MOESM2_ESM.docx]

Supplemental Table 2. Bivariate associations between risk of incident arrest and respondent characteristics, restraining order service, linkage to firearms, and firearm recovery

| Order-Recovery Status and Respondent Characteristic | Risk of Incident Arrest | | | | | | | |
| --- | --- | --- | --- | --- | --- | --- | --- | --- |
|  | Any Offense | | Offenses Not Involving Violence or Firearms | | Domestic Violence | | Other Violent or Firearm-Related Offenses | |
|  | OR  (95% CI) | p Value | OR  (95% CI) | p Value | OR  (95% CI) | p Value | OR  (95% CI) | p Value |
| Sex | | | | | | | | |
| Male (n=2,439) | 1.6 (1.2-2.1) | 0.0003 | 1.6 (1.2-2.1) | 0.0014 | 1.6 (1.1-2.4) | 0.0164 | 2.4 (1.5-3.9) | 0.0002 |
| Female (n=533) | Referent |  | Referent |  | Referent |  | Referent |  |
| Age^a^, years | | | | | | | | |
| ≤24 (n=418) | 1.7 (1.2-2.2) | 0.0004 | 1.6 (1.2-2.2) | 0.0005 | 1.4 (0.9-2.2) | 0.1162 | 1.8 (1.2-2.8) | 0.0281 |
| 25-3 (n=807) | 1.6 (1.2-2.0) |  | 1.7 (1.3-2.2) |  | 1.6 (1.1-2.2) |  | 1.7 (1.1-2.4) |  |
| 35-44 (n=850) | 1.3 (1.0-1.6) |  | 1.3 (1.0-1.7) |  | 1.3 (0.9-1.8) |  | 1.4 (1.0-2.1) |  |
| ≥45 (n=821) | Referent |  | Referent |  | Referent |  | Referent |  |
| Prior arrest charges | | | | | | | | |
| 10+ (n=496) | 59.2 (43.2-81.2) | <0.0001 | 67.0 (46.6-96.5) | <0.0001 | 16.9 (11.3-25.3) | <0.0001 | 50.4 (29.2-87.2) | <0.0001 |
| 3-9 (n=378) | 19.3 (14.0-26.7) |  | 23.3 (15.9-34.0) |  | 11.8 (7.6-18.3) |  | 18.3 (10.1-33.2) |  |
| 1-2 (n=299) | 9.1 (6.3-13.1) |  | 11.1 (7.3-17.1) |  | 4.6 (2.7-8.0) |  | 13.4 (7.0-25.4) |  |
| None (n=1,799) | Referent |  | Referent |  | Referent |  | Referent |  |
| Time from most recent arrest charge to date of restraining order ^a^, years | | | | | | | | |
| 0-5 (n=953) | 3.2 (2.3-4.5) | <0.0001 | 3.7 (2.6-5.4) | <0.0001 | 2.3 (1.4-3.7) | 0.0008 | 2.4 (1.5-3.9) | 0.0003 |
| 6+ (n=220) | Referent |  | Referent |  | Referent |  | Referent |  |
| Pre-existing firearms prohibition^b^ | | | | | | | | |
| Y (n=578) | 2.4 (1.9-3.0) | <0.0001 | 2.4 (1.9-3.1) | <0.0001 | 1.5 (1.1-2.0) | 0.0161 | 1.8 (1.3-2.4) | 0.0003 |
| N (n=595) | Referent |  | Referent |  | Referent |  | Referent |  |
| Order served | | | | | | | | |
| Y (n=1,677) | 1.3 (1.1-1.6) | 0.0013 | 1.3 (1.1-1.6) | 0.0038 | 1.7 (1.3-2.3) | 0.0002 | 1.3 (1.0-1.8) | 0.0428 |
| N (n=1,295) | Referent |  | Referent |  | Referent |  | Referent |  |
| Linked to firearms | | | | | | | | |
| Y (n=525) | 1.0 (0.8-1.3) | 0.9275 | 1.0 (0.8-1.3) | 0.8281 | 0.9 (0.6-1.3) | 0.4641 | 1.1 (0.8-1.6) | 0.6188 |
| N (n=2,447) | Referent |  | Referent |  | Referent |  | Referent |  |
| Firearms recovered^c^ | | | | | | | | |
| Y (n=119) | 0.4 (0.2-0.8) | 0.0075 | 0.3 (0.1-0.7) | 0.006 | 1.0 (0.5-2.4) | 0.9264 | 0.5 (0.2-1.3) | 0.1635 |
| N (n=242) | Referent |  | Referent |  | Referent |  | Referent |  |
| Follow-up time, per month | | | | | | | | |
|  | 1.0 (1.0-1.0) | 0.0007 | 1.0 (1.0-1.0) | <0.0001 | 1.0 (1.0-1.0) | 0.1197 | 1.0 (1.0-1.0) | 0.0008 |

^a^ Age was missing for 66 individuals

^b^ Results are for respondents with prior arrests.

^c^ Results are for 361 respondents who were linked to firearms and whose orders were served.
