# Supplementary material for: Firearms and the incidence of arrest among respondents to domestic violence restraining orders
Source: Inj Epidemiol. 2015 Jun 23;2(1):14. doi: 10.1186/s40621-015-0047-2 (PMC5005597; doi:10.1186/s40621-015-0047-2)
Supplement: Additional file 3: Table S3. — Sensitivity analysis. [file 40621_2015_47_MOESM3_ESM.docx]

Supplemental Table 3. Sensitivity analysis. Multivariate associations between risk of incident arrest and respondent characteristics, prior criminal history, restraining order service, and linkage to firearms: subjects not screening positive for access to firearms but having a prior criminal history involving firearms recoded as having access to firearms^a^.

| Order-Recovery Status and Respondent Characteristic | Risk of Incident Arrest | | | | | | | |
| --- | --- | --- | --- | --- | --- | --- | --- | --- |
|  | Any Offense | | Offenses Not Involving Violence or Firearms | | Domestic Violence | | Other Violent or Firearm-Related Offenses | |
|  | OR  (95% CI) | p Value | OR  (95% CI) | p Value | OR  (95% CI) | p Value | OR  (95% CI) | p Value |
| Sex | | | | | | | | |
| Male (n=2,439) | 1.0 (0.7-1.4) | 0.8514 | 1.0 (0.7-1.4) | 0.8823 | 1.2 (0.8-1.8) | 0.4905 | 1.7 (1.0-2.8) | 0.0518 |
| Female (n=533) | Referent |  | Referent |  | Referent |  | Referent |  |
| Age^b^, years | | | | | | | | |
| ≤24 (n=418) | 2.7 (1.9-4.0) | <0.0001 | 2.5 (1.6-3.7) | <0.0001 | 1.5 (0.9-2.4) | 0.1890 | 2.5 (1.5-4.1) | 0.0021 |
| 25-34 (n=807) | 1.5 (1.1-2.0) |  | 1.6 (1.1-2.2) |  | 1.3 (0.9-2.0) |  | 1.4 (0.9-2.2) |  |
| 35-44 (n=850) | 1.0 (0.7-1.3) |  | 1.0 (0.7-1.4) |  | 1.0 (0.7-1.5) |  | 1.1 (0.8-1.7) |  |
| ≥45 (n=831) | Referent |  | Referent |  | Referent |  | Referent |  |
| Prior arrest charges | | | | | | | | |
| 10+ (n=496) | 72.2 (51.6-101.1) | <0.0001 | 79.4 (54.2-116.2) | <0.0001 | 18.0 (11.9-27.2) | <0.0001 | 51.7 (29.6-90.3) | <0.0001 |
| 3-9 (n=378) | 23.2 (16.5-32.7) |  | 27.9 (18.8-41.4) |  | 13.0 (8.3-20.4) |  | 18.7 (10.2-34.3) |  |
| 1-2 (n=299) | 11.6 (7.9-17.0) |  | 14.3 (9.2-22.3) |  | 5.3 (3.0-9.3) |  | 15.4 (8.0-29.8) |  |
| None (n=1799) | Referent |  | Referent |  | Referent |  | Referent |  |
| Time from most recent arrest charge to date of restraining order ^c^, years | | | | | | | | |
| 0-5 (n=953) | 1.8 (1.2-2.6) | 0.0018 | 2.1 (1.4-3.1) | 0.0002 | 1.5 (0.9-2.6) | 0.0901 | 1.5 (0.9-2.5) | 0.1335 |
| 6+ (n=220) | Referent |  | Referent |  | Referent |  | Referent |  |
| Order served | | | | | | | | |
| Y (n=1,677) | 1.2 (1.0-1.5) | 0.0940 | 1.2 (0.9-1.5) | 0.1376 | 1.6 (1.2-2.2) | 0.002 | 1.2 (0.9-1.6) | 0.3228 |
| N (n=1,295) | Referent |  | Referent |  | Referent |  | Referent |  |
| Linked to firearms | | | | | | | | |
| Y (n=607) | 0.8 (0.6-1.0) | 0.0658 | 0.8 (0.6-1.0) | 0.1029 | 0.6 (0.4-0.8) | 0.0028 | 0.9 (0.7-1.3) | 0.7068 |
| N (n=2,365) | Referent |  | Referent |  | Referent |  | Referent |  |
| Follow-up time, per month | | | | | | | | |
|  | 1.0 (1.0-1.0) | <0.0001 | 1.0 (1.0-1.1) | <0.0001 | 1.0 (1.0-1.0) | 0.0045 | 1.0 (1.0-1.0) | <0.0001 |

^a^ Model includes all variables in table.

^b^ Age was missing for 66 individuals.

^c^ Results are for respondents with prior arrests. Pre-existing firearm prohibition status and number of prior arrest charges were highly correlated and could not both be entered into the model.
